# Low-Dimensional Synergistic Representation of Bilateral Reaching Movements

**DOI:** 10.3389/fbioe.2017.00002

**Published:** 2017-02-10

**Authors:** Martin K. Burns, Vrajeshri Patel, Ionut Florescu, Kishore V. Pochiraju, Ramana Vinjamuri

**Affiliations:** ^1^Sensorimotor Control Laboratory, Department of Biomedical Engineering, Chemistry, and Biological Sciences, Stevens Institute of Technology, Hoboken, NJ, USA

**Keywords:** motor control, kinematic synergies, bilateral upper limb movements, activities of daily living, principal component analysis

## Abstract

Kinematic and neuromuscular synergies have been found in numerous aspects of human motion. This study aims to determine how effectively kinematic synergies in bilateral upper arm movements can be used to replicate complex activities of daily living (ADL) tasks using a sparse optimization algorithm. Ten right-handed subjects executed 18 rapid and 11 natural-paced ADL tasks requiring bimanual coordination while sitting at a table. A position tracking system was used to track the subjects’ arms in space, and angular velocities over time for shoulder abduction, shoulder flexion, shoulder internal rotation, and elbow flexion for each arm were computed. Principal component analysis (PCA) was used to generate kinematic synergies from the rapid-paced task set for each subject. The first three synergies accounted for 80.3 ± 3.8% of variance, while the first eight accounted for 94.8 ± 0.85%. The first and second synergies appeared to encode symmetric reaching motions which were highly correlated across subjects. The first three synergies were correlated between left and right arms within subjects, whereas synergies four through eight were not, indicating asymmetries between left and right arms in only the higher order synergies. The synergies were then used to reconstruct each natural-paced task using the *l*_1_-norm minimization algorithm. Temporal dilations of the synergies were introduced in order to model the temporal scaling of movement patterns achieved by the cerebellum and basal ganglia as reported previously in the literature. Reconstruction error was reduced by introducing synergy dilations, and cumulative recruitment of several synergies was significantly reduced in the first 10% of training task time by introducing temporal dilations. The outcomes of this work could open new scenarios for the applications of postural synergies to the control of robotic systems, with potential applications in rehabilitation. These synergies not only help in providing near-natural control but also provide simplified strategies for design and control of artificial limbs. Potential applications of these bilateral synergies were discussed and future directions were proposed.

## Introduction

The human arm is a highly complex structure with an equally sophisticated control system. Each arm possesses 11 independent degrees of freedom (DoF) defined from the pectoral girdle to the wrist, which are actuated by approximately 32 muscles (Mackenzie and Iberall, 1994). The brain, therefore, has to coordinate over 60 different controls in order to operate both arms, yet accomplishes this task with apparent ease. How the brain handles real-time control of two redundant, high DoF manipulators during activities of daily living (ADL) is known as the degrees-of-freedom (DoF) problem (Bernstein, 1967; Latash et al., 2007) and is the subject of much research, including the present work. Progress in this field has applications in numerous areas including motor rehabilitation, assistive and prosthetic technology, and robotic control.

Evidence suggests that the brain may control the limbs by scaling, offsetting, and temporally dilating fundamental movements encoded in the sensorimotor system (Viviani and Terzuolo, 1980; Brooks, 1986). Previous research has shown that the brain executes tasks by using certain movement patterns while preserving their relative spatiotemporal proportions. Viviani and Terzuolo (1980) have shown in handwriting tasks that increased letter size still results in similar execution times by automatically increasing writing speed. Furthermore, slowing down a writing task results in temporal dilation of a common velocity pattern, preserving the relative occurrence of velocity profile features in time while reducing velocity amplitude (Brooks, 1986).

These patterns of motion have been developed into the concept of synergies, which can be defined as “a collection of relatively independent degrees of freedom that behave as a single functional unit” (Turvey, 2007). Synergies exist in either the joint angular velocity space, in the form of kinematic synergies, or neuromuscular activity space, in the form of neuromuscular synergies. Linear discriminant analysis, singular value decomposition (SVD), principal component analysis (PCA), non-negative matrix factorization (NMF), artificial neural networks, and many other algorithms have been used in the literature to derive synergies for hand grasps, gait patterns, and single-arm motion (Merckle et al., 1998; Santello et al., 1998; Vinjamuri et al., 2010; Roh et al., 2013; Alibeji et al., 2015). NMF is typically used to derive neuromuscular synergies (Tresch and Jarc, 2009), while PCA is frequently used to derive kinematic synergies as in Mason et al. (2001) and Vinjamuri et al. (2010). PCA-derived kinematic synergies have been demonstrated to perform favorably when directly compared to those from other linear and non-linear dimensionality reduction methods when applied to hand grasp reconstruction (Patel et al., 2015a).

Recent work has been aimed at integrating synergies into the control of robotic systems with the goal of producing a simplified control scheme for high DoF devices. The authors in Chen et al. (2015) have demonstrated an anthropomorphic robotic hand that has two mechanically implemented postural synergies which could successfully grasp various objects. Several groups have also proposed autonomous, control systems for high DoF robotic and virtual hands based on two postural synergies (Wimbock et al., 2011) and four postural synergies (Rosell et al., 2011; Segil and Weir, 2013), whereas Matrone et al. (2012) have demonstrated real-time myoelectric control of a robotic hand using two postural synergies with able-bodied subjects. An EMG-based control scheme was also introduced by Artemiadis and Kyriakopoulos (2010), which controls a 7-DoF robotic arm using kinematic and muscular synergies. The review recently published by Santello et al. (2016) gives a thorough description of the state of the art concerning dexterous hand control using synergies and highlights some future directions merging synergies with compliant design. Synergies derived using NMF have also been applied to optimal movement generation for virtual arms (Fu et al., 2013) as well as myocontrol of a multi-DoF planar robotic arm using muscle synergies (Lunardini et al., 2015). So far, work has been focused on using time-invariant postural synergies in the kinematic domain and restricted to unimanual processes.

Bilateral spatiotemporal kinematic synergies such as those presented here may be used as the controlled variable in future robotic systems that can be manipulated using EMG, EEG, or some other biosignal input. Whereas postural/spatial synergies attempt to linearize joint motion relative to each other, a time-varying approach allows more flexibility to capture the non-linear behaviors inherent to complex systems. An open question for such a system is whether or not ADL tasks are within the “workspace” of a system that is only manipulated using time-varying kinematic synergies. In other words, is it possible to manipulate bilateral spatiotemporal kinematic synergies by scaling their amplitudes and temporal offsets in such a way as to replicate ADL-like tasks.

In this study, we derive spatiotemporal kinematic synergies from rapidly paced ADL tasks for 10 able-bodied subjects. Tasks that require coordination of both arms and can be classified as symmetric in-phase, symmetric out-of-phase, asymmetric, or coupled are chosen. PCA is used to derive time-varying kinematic synergies from eight joint angular velocity profiles across both arms recorded during these rapid tasks. A separate set of tasks performed at a natural pace are reconstructed using the *l*_1_*-*norm minimization algorithm to select optimal amplitudes and temporal offsets and dilations of these synergies. The derived synergies are characterized in terms of intersubject and interlimb correlations, accuracy of reconstruction, and trends in their recruitment levels throughout the task duration.

## Subjects and Methods

The present study was conducted under IRB Approved Protocol # 2014-026/2015-022 at the Stevens Institute of Technology. Ten subjects were recruited in the study after obtaining written informed consent. Subjects performed ADL-like tasks while their movements were recorded using an electromagnetic motion tracking system (Polhemus LIBERTY). Positional data from each sensor were converted into joint angles, synergies were derived using PCA in the joint angle velocity domain, and a separate set of tasks were reconstructed from the derived synergies using the *l*_1_-norm minimization algorithm.

### Data Capture

An electromagnetic tracking system (Polhemus LIBERTY, TX4 source) was used to record positional data of the subject during each task using their proprietary software (PiMgr). The study was executed in a minimal-metal environment with a compensation map calibration executed monthly to account for disturbances due to metal in the construction of the room. The workspace was calibrated such that the origin was on the edge of the table, centered in front of the subject. Positive Z extended upwards toward the ceiling, positive X extended forward away from the subject, and positive Y extended to the subject’s left. Data were captured at 240 Hz and filtered using a 3 Hz fourth-order low-pass Butterworth filter.

### Kinematic Model

Several groups have developed refined anatomical models with the intent of capturing kinematic data from subjects as they perform tasks. Most of this work has addressed hurdles using optical systems such as 3D interpolation of one or multiple 2D viewpoints (Sidenbladh et al., 2000; Chen et al., 2010), and soft tissue deformation (Gabiccini et al., 2013). Under guidance from Wu et al. (2005), this work utilized an electromagnetic tracking system to capture the positions of several convenient landmarks on the torso, left, and right arms with a positional accuracy of approximately 2.5 mm in *x*, *y*, and *z*. The tracking system lacks line of sight issues and readily supplies Cartesian positions of these landmarks.

**Figure 1:**
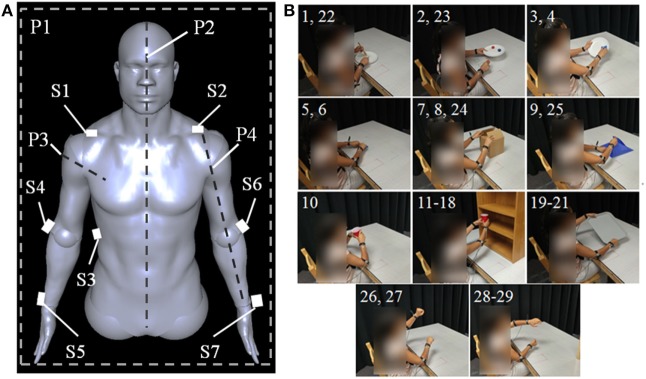
**(A)** Sensor placement on body. *S*_1_ and *S*_2_ are positioned at the lateral head of the clavicles, S3 is placed on the right side on the body’s midaxillary line, *S*_4_ and *S*_6_ are placed on the outer side of the elbow, and *S*_5_ and *S*_7_ are placed on the wrists between the distal heads of the radius and ulna. *P*_1_ indicates the coronal plane, *P*_2_ indicates the sagittal plane, *P*_3_ indicates the plane normal to *V*_ae_, and *P*_4_ indicates the plane containing *V*_ae_ and *V*_aw_. *P*_3_ and *P*_4_ exist for both the left and right arms. **(B)** Tasks executed during study. Each panel is labeled with a number corresponding to the task in Table 2 which is shown. Subjects start each task with their hands in the rest positions marked by the visible red boxes.

Seven sensors were placed on the body as shown in Figure 1A. Three sensors defined the trunk of the subject, while two additional sensors per arm tracked elbow and wrist movements in space. *S*_1_ and *S*_2_ were placed at the lateral head of the clavicle on the subject’s right and left shoulder, respectively, while *S*_3_ was placed on the subject’s right side near the middle of the rib cage on the midaxillary line. *S*_4_ and *S*_6_ were placed on the lateral side of the subject’s elbows over the joint’s center of rotation. *S*_5_ and *S*_7_ were placed on the dorsal side of the subject’s wrists and were centered between the distal ulnar and radial heads. The filtered *X*, *Y*, and *Z* trajectories captured by the tracking system were converted to joint angles as follows.

Three shoulder angles and one elbow angle were calculated for each side of the subject: shoulder abduction/adduction, flexion/extension, and internal/external and elbow flexion/extension. These angles are calculated using six vectors: *V*_should_, *V*_side_,*V*_ae_, and *V*_aw_, where *a* = *L, R* to indicate the left or right arm. *V*_should_ is a vector from the subject’s left shoulder to their right shoulder sensors, *V*_side_ is a vector from the right shoulder sensor to the sensor on the right side of the torso, *V*_ae_ is a vector from the shoulder to the elbow on each respective side, and *V*_aw_ is the vector from the elbow to the wrist on each respective side. These vectors were calculated as:

$$S_{i}=\left[\begin{array}{c} x_{i}\\ y_{i}\\ z_{i} \end{array}\right]$$

$$V_{\text{should}}=S_{1}-S_{2}$$

$$V_{\text{side}}=S_{1}-S_{3}$$

$$V_{\text{Re}}=S_{1}-S_{4},\;V_{\text{Le}}=S_{2}-S_{6}$$

$$V_{\text{Rw}}=S_{4}-S_{5},\;V_{\text{Lw}}=S_{6}-S_{7}$$

Abduction/adduction was found by first projecting the elbow vector, *V*_ae_, onto the coronal plane:

(1) $$V_{\text{ae,proj}}=V_{\text{ae}}-\left(V_{\text{ae}}\cdot n_{c}\right)\frac{n_{c}}{∥n_{c}∥}$$

where *n_c_* is the vector normal to the coronal plane and is defined as the cross product between *V*_should_ and *V*_side_. The shoulder abduction angle, θ_sa_, between *V*_ae,proj_ and *V*_should_ was found using

(2) $$\theta_{\text{sa}}=\cos^{-1}\frac{V_{\text{should}}\cdot V_{\text{ae,proj}}}{∥V_{\text{should}}∥∥V_{\text{ae,proj}}∥}$$

The flexion/extension angle was found in the same manner as in Eqs 1 and 2 except by projecting the vectors onto the sagittal plane using *n_s_*, the normal vector to the sagittal plane, which was found by normalizing *V*_should_. The internal/external rotation of the upper arm was found by projecting the wrist vector, *V*_aw_, and *V*_should_ onto the plane normal to *V*_ae_ and calculating the angle between the two projections as in Eq. 2. Elbow flexion/extension was calculated using *V*_aw_ and *V*_ae_ in Eq. 2. Sign changes were determined by comparing the vector cross product to the corresponding normal vector on the plane. Positive angles correspond to shoulder abduction, shoulder flexion, shoulder external rotation, and elbow flexion. These joint angle calculations were performed offline using a MATLAB function to get joint angle trajectories over time, and the resulting motion profiles were differentiated to get joint angular velocities.

#### Model Validation

Table 1 shows the accuracy of the joint angle calculations reported here, compared to goniometer measurements. An iGaging 7^″^ goniometer (Anytime Inc., Los Angeles, CA, USA) with a precision of 0.1° and resolution of 0.05° was used to enforce the measured (ground truth) readings presented in Table 1. Each of the eight joints (two angles per joint, indicating minimum and maximum angles) were measured independently in each trial. Sensor positions were recorded and filtered using PiMgr (see Data Capture) in 2 s recordings. Three repetitions were captured for each trial. Joint angles were calculated from the time-series position data using our model to yield 2-s long joint angle postures, which were then averaged across the 2 s to get a joint angle for each repetition of each trial. Mean and SD for the calculated angles shown in Table 1 are computed across repetitions. The difference between the model-estimated joint angles and the ground truth measured by goniometers for minimum and maximum angles is 8.8° and 8.6°, respectively. Error normalized to ground truth measurements reveals a mean of 36% error for the minimum measurement and 10% error for the maximum measurement, in large part because the minimum angle is smaller in magnitude than the maximum. Since reported results are in the velocity domain a large part of this error is negated in differentiation.

**Table 1:** **Computation of normalized error by the difference between measured (by goniometer) and estimated (by the model) joint angles (in degrees, mean ± SD)**.

	Minimum angle (°)			Maximum angle (°)		
Joints	Measured by goniometer	Estimated by the model	Normalized error (%)	Measured by goniometer	Estimated by the model	Normalized error (%)
R. Should. Abd.	30	30.8 ± 1.42	3.4	90	85.2 ± 1.41	5.3
R. Should. Flex	30	34.3 ± 1.55	14.3	90	75.8 ± 1.54	15.8
R. Should. Int.	−45	−33.1 ± 3.05	26.6	45	45.4 ± 1.35	0.8
R. Elbow Flex	20	40.4 ± 2.49	101.9	90	99.2 ± 2.61	10.2
L. Should. Abd.	30	29.8 ± 1.15	3	90	78.4 ± 2.71	12.9
L. Should. Flex	30	33.8 ± 1.90	12.7	90	69.9 ± 2.74	22.3
L. Should. Int.	−45	−37.5 ± 6.27	16.6	45	40.9 ± 6.85	4.7
L. Elbow Flex	20	41.4 ± 1.82	107.2	90	94.1 ± 2.67	4.6

### Subjects

Subjects were recruited, with written and informed consent, based on the criteria specified in the approved IRB. Healthy subjects with no history of right or left upper limb injury or weakness and no cognitive or motor impairments were allowed in the study after signing a consent form and filling out a basic medical questionnaire. Ten subjects of age 18–25 years were recruited (mean 20); of which 4 were female and 10 were self-reported right-hand dominant.

### Experiment Procedure

Upon arrival subjects were fitted with hook-and-loop harnesses for the position sensors. Straps were adjusted so the sensors were held firmly in place without impeding the subject’s motion. Basic range of motion exercises were performed to ensure that all straps and wires were settled and that the sensors remained in the proper locations. Subjects first executed 18 rapid training tasks, during which they were instructed to execute each task as quickly as they could successfully be completed. Three repetitions of each task were completed before performing the next task in the same fixed order for every subject. Eleven testing tasks were then performed in a fixed order at the subject’s natural pace with three repetitions each. Each session lasted approximately 90 min with a short break offered between task phases.

Figure 1B and Table 2 show each of the tasks performed in the study. These tasks were selected based on Barreca et al. (2005) and Foti and Koketsu (2013) as a cross-section of what is performed during ADL while requiring coordinated motion of both arms and being executable in the testing environment. These tasks were grouped into four categories to describe their type of motion: symmetric in-phase, symmetric out-of-phase, asymmetric, and coupled. Symmetric in-phase motions involve mirror symmetry between the two arms. This symmetry is typically about the midline but can be present in any direction. Symmetric out-of-phase motions have the same mirror symmetry as the in-phase category, except the motions of the left and right arms are offset in time. Asymmetric motions involve no symmetry between the two arms: each arm executes a different motion trajectory from the other such as washing a dish or using a fork and knife. Coupled motions involve both arms manipulating one object, such as when moving a box or tray, which results in a fixed relationship between the endpoints of each arm. Table 3 shows the number of each type of task present in the training and testing phase. Subjects were given instruction on what to do in each task with special care taken not to coach how to execute the motions. Subjects began each task with their hands in a flat resting position marked with red rectangles. Subjects were instructed to stop at the end of the task without returning to the rest position.

**Table 2:** **Task list as executed in experiment**.

Experimentphase	Task number	**Task**	**Category**
Training tasks	1	Knife and fork	Asymmetric
	2	Pick object off plate	Asymmetric
	3–4	Scrub dish	Asymmetric
	5–6	Scrub table	Coupled
	7–8	Open box	Symmetric in-phase
	9	Fold clothes	Symmetric out-of-phase
	10	Drink from cup	Coupled
	11–14	Place cup on shelf	Coupled
	15–18	Pick cup off shelf	Coupled
Testing tasks	19–21	Manipulate tray	Coupled
	22	Knife and fork	Asymmetric
	23	Pick object off plate	Asymmetric
	24	Open box	Symmetric in-phase
	25	Fold clothes	Symmetric out-of-phase
	26–27	Pretend steering wheel	Asymmetric
	28–29	Pretend ladder climb	Symmetric out-of-phase

**Table 3:** **Summary of task categories**.

Task category	Number of training tasks	Number of testing tasks
Symmetric in-phase	2	1
Symmetric out-of-phase	1	3
Asymmetric	4	4
Coupled	11	3

Tasks 1 and 22, the knife and fork task, involved the subject picking up a knife and fork, positioning them on a plate as if to cut food, and executing a cutting motion with the knife. Task 1 involved one cutting motion and task 22 involved three. Tasks 2 and 23, picking object off plate, involved the subject picking up a plate holding a small wooden object with their non-dominant hand and using their dominant hand to pick the object off the plate and place it on a target marked on the table. Tasks 3 and 4, scrub dish, involved the subject picking up a plate in their non-dominant hand and using a sponge with their dominant hand to wipe the plate in a circular clockwise (task 3) or counterclockwise (task 4) motion for one complete cycle. Tasks 5 and 6, scrub table, consist of the subject reaching for a sponge and wiping it in a shallow upright (task 5) or upside down (task 6) V pattern along the surface of the table.

The open box task, number 7, 8, and 24, involved the subject simultaneously opening the left and right flaps (task 7), the front and back flaps (task 8), or both sets of flaps sequentially (task 27) of a medium shipping box. Tasks 9 and 25, the fold clothes task, consist of identical motions between the training and testing set. The subject grasped a large piece of cloth along the outer edges and folded the left and right thirds over the center.

Task 10, drink from cup, consisted of the subject grasping a weighted cup placed in front of them with both hands and raising it up to their mouth as if to drink. Tasks 11–14, place cup on shelf, involved the subject picking up a weighted cup with both hands and placing it on one of four locations on a small set of shelves (top left, top right, bottom left, and bottom right sections of shelf for tasks 11–14, respectively). Tasks 15–18, pick cup off shelf, has the same sequence as 11–14 except the subject moves the cup from the shelf to the table.

Tasks 19–21, manipulate tray, involved the subject picking up a tray while tilting it to the left (task 19), right (task 21), or not tilting (task 20). Tasks 26 and 27, imagined steer wheel, involved the subject pantomiming a steering motion. Subjects were instructed to pretend to turn a steering wheel in a hand-over-hand fashion in either the clockwise direction (task 26) or the counterclockwise direction (task 27) for three complete cycles. Tasks 28 and 29, imagined ladder climb, involved the subject pantomiming climbing up (task 28) and down (task 29) a ladder while seated.

The measured velocity trajectories were windowed using a threshold of 5% maximum repetition velocity to identify task start/end. Windowed training tasks were then averaged across repetitions for each subject to produce 18 windowed, filtered, averaged joint angular velocity profiles for synergy derivation. Testing task data were converted to joint angular velocity as above and were windowed to task onset without averaging across repetitions. Testing tasks were only windowed to task start to ensure that task duration always exceeded synergy duration.

### Synergy Derivation

Table 4 documents all symbols used in this section for reference. The measured velocity profiles for the training tasks were formatted into a matrix for each subject during data processing with *J* rows, *T*_max_ columns, and *N* pages. For the training tasks, the study used *J* = 8 joints, *T*_max_ = number of samples in the longest windowed training task, and *n* training tasks with *N* = 18 total.

$$V_{n}=\;\left[\begin{array}{ccc} v_{1}^{n}\left(1\right) & \ldots & v_{1}^{n}\left(T_{\text{max}}\right)\\ \vdots & \ddots & \vdots \\ v_{J}^{n}\left(1\right) & \ldots & v_{J}^{n}\left(T_{\text{max}}\right) \end{array}\right]=\left[\begin{array}{ccc} v_{1}^{n}\left(1\right) & \ldots & v_{1}^{n}\left(T_{\text{max}}\right)\\ \vdots & \ddots & \vdots \\ v_{8}^{n}\left(1\right) & \ldots & v_{8}^{n}(T_{\text{max}}\text{)} \end{array}\right]$$

**Table 4:** **Definitions of variables used in Section “Materials and Methods**.”

Symbol	Definition	Maximum value
*J*	Index for joint number	*J* = 8
*T*	Index for task sample	*T*_max_ (training tasks), *T_d_* (downsampled testing tasks)
*n*	Index for training task number	*N* = 18
*m*	Index for synergies	*M* = 8
*l*	Index for testing task number	*L* = 11
*D*	Index for temporal synergy dilations	*D* = 3
*T_s,D_*	Length of *D*th dilation of *m*th synergy	–

Tasks were padded with zeros on the end to reach *T*_max_ length. *T*_max_ varies for each subject and ranged from 400 to 608 samples. In order to extract spatiotemporal synergies using PCA, the 8 × *T*_max_ dimensional velocity matrix *V_n_* for a given task is manipulated into a row vector, ${\bar{V}}_{n}$, with *J⋅T*_max_ columns where each instant of time is represented as an additional set of *J* joints. Each task’s row vector is concatenated into an *N* × *J⋅T*_max_ task matrix, *V*:

$$V=\left[\begin{array}{c} {\bar{V}}_{1}\\ {\bar{V}}_{2}\\ \vdots \\ {\bar{V}}_{n} \end{array}\right]=\left[\begin{array}{c} \left[\begin{array}{ccccccc} v_{1}^{1}\left(1\right) & \cdots & v_{8}^{1}\left(1\right) & \cdots & v_{1}^{1}\left(T_{\text{max}}\right) & \cdots & v_{8}^{1}\left(T_{\text{max}}\right) \end{array}\right]\\ \left[\begin{array}{ccccccc} v_{1}^{2}\left(1\right) & \cdots & v_{8}^{2}\left(1\right) & \cdots & v_{1}^{2}\left(T_{\text{max}}\right) & \cdots & v_{8}^{2}\left(T_{\text{max}}\right) \end{array}\right]\\ \vdots \\ \left[\begin{array}{ccccccc} v_{1}^{N}\left(1\right) & \cdots & v_{8}^{N}\left(1\right) & \cdots & v_{1}^{N}\left(T_{\text{max}}\right) & \cdots & v_{8}^{N}\left(T_{\text{max}}\right) \end{array}\right] \end{array}\right]$$

Principal component analysis operates on this matrix as though it were a *J⋅T*_max_-joint system observed at *N* instants of time, effectively treating the time-series velocity profiles as a single instant of a *J⋅T*_max_-joint system. The task matrix is resolved into three-component matrices using SVD:

V=U∑S

where *U* is an *N* × *N* square matrix with orthogonal columns, Σ is an *N* × *J⋅T*_max_ diagonal matrix, and *S* is a *J⋅T*_max_ × *J⋅T*_max_ square matrix with orthogonal rows. The diagonal elements of Σ correspond to the singular values, λ, of *V*.

$$\sum =\text{diag}\{{\text{λ}}_{1},{\text{λ}}_{2},\;\ldots ,\;{\text{λ}}_{N}\}$$

In this form, the first *m* rows of *S* correspond to the first *m* principal components, or synergies, where *m* < *N*:

$$S=\;\left[\begin{array}{ccccccc} s_{1}^{1}\left(1\right) & \cdots & s_{J}^{1}\left(1\right) & \cdots & s_{1}^{1}\left(T_{\text{max}}\right) & \cdots & s_{J}^{1}\left(T_{\text{max}}\right)\\ s_{1}^{2}\left(1\right) & \cdots & s_{J}^{2}\left(1\right) & \cdots & s_{1}^{2}\left(T_{\text{max}}\right) & \cdots & s_{J}^{2}\left(T_{\text{max}}\right)\\ & & & \vdots & & & \\ s_{1}^{M}\left(1\right) & \cdots & s_{J}^{M}\left(1\right) & \cdots & s_{1}^{M}\left(T_{\text{max}}\right) & \cdots & s_{J}^{M}\left(T_{\text{max}}\right)\\ & & & \vdots & & & \\ s_{1}^{N}\left(1\right) & \cdots & s_{J}^{N}\left(1\right) & \cdots & s_{1}^{N}\left(T_{\text{max}}\right) & \cdots & s_{J}^{N}(T_{\text{max}}\text{)} \end{array}\right]$$

*U*, Σ, and *S* are computed from *V* using the SVD function in MATLAB. The original *V* matrix can be approximated as $\;\tilde{\;V}$ by isolating the first *M* columns of *U*, *M* × *M* elements of Σ, and *M* rows of *S*. The matrix *U_M_*diag{λ*_1_*, λ*_2_*,… λ*_M_*} is now denoted as the weight matrix for the *n*th task and first *M* synergies for *S_M_*:

(3) $$V\approx \;\tilde{\;V}=U_{M}\text{diag}\left\{\lambda_{1},\lambda_{2},\ldots ,\lambda_{M}\right\}S_{M}=W_{M}^{n}S_{M}$$

It follows from Eq. 3 that the time-series components of $\;\tilde{\;V}$ over time can be expressed as a weighted sum of *M* principal components:

(4) $${\tilde{\;v}}_{j}^{n}\left(t\right)=\sum_{m=1}^{M}w_{m}^{n}s_{j}^{m}(t)$$

Since the singular values found in Σ are related to the spread of data along each principle axis, i.e., variance in that direction, an index known as fraction of sum-squared variance can be calculated from the diagonal elements of Σ. This index describes the fraction of total variance accounted for by the first *m* synergies and is useful as an indicator of how many principal components are needed to adequately represent the data.

(5) $$\frac{\lambda_{1}^{2}+\lambda_{2}^{2}+...+\lambda_{M}^{2}}{\lambda_{1}^{2}+\lambda_{2}^{2}+...+\lambda_{N}^{2}}$$

An index threshold of 95% variance is used to determine how many synergies, *M*, are required to represent the training task data.

Principal component analysis assumes stationary input variables, but the input data contain time-varying joint angular velocities. Here, “time-varying” refers to a motion consisting of a sequence of postures that change with time. However, the statistical properties of the synergies are quite stationary. PCA also assumes independent and identically distributed variables, but the input data that contain bilateral arm postures are not strictly independent (as there are biomechanical constraints that lead to joint correlations) as is the case with many real-world variables. PCA is a non-parametric method, i.e., it does not require any prior knowledge. Although this makes the application of this method simple, the method itself assumes linearity, which could be a weakness in many applications. We have compared the performance of PCA with other non-linear methods in Patel et al. (2015a) and found that PCA outperformed other methods. Using this exploratory analysis has previously led us to anatomically informing and meaningful synergies as principal components. These synergies could represent 100 postural movements with as low as six synergies with accuracy greater than 90% (Vinjamuri et al., 2010). These synergies also showed the effect on visual and tactile feedback in reaching and grasping movements (Patel et al., 2015b).

### Reconstruction

Before performing reconstruction, the synergies and testing data were downsampled from 240 to 60 Hz due to the relatively long duration of synergies and testing tasks leading to excessive computation time. The testing task matrix, *R*, was reformatted from a *J* × *T_d_* × *L* matrix for *J* joints, *T_d_* samples in the downsampled task, and *L* = 11 tasks into a *J⋅T_d_* row, *L* column 2D matrix with each column defined as a separate task:

$$R=\left[\begin{array}{cccc} \left[\begin{array}{c} r_{1}^{1}\left(1\right)\\ \vdots \\ r_{j}^{1}\left(1\right)\\ \vdots \\ r_{1}^{1}\left(T_{d}\right)\\ \vdots \\ r_{j}^{1}\left(T_{d}\right) \end{array}\right], & \left[\begin{array}{c} r_{1}^{2}\left(1\right)\\ \vdots \\ r_{j}^{2}\left(1\right)\\ \vdots \\ r_{1}^{2}\left(T_{d}\right)\\ \vdots \\ r_{j}^{2}\left(T_{d}\right) \end{array}\right], & \cdots & \left[\begin{array}{c} r_{1}^{L}\left(1\right)\\ \vdots \\ r_{j}^{L}\left(1\right)\\ \vdots \\ r_{1}^{L}\left(T_{d}\right)\\ \vdots \\ r_{j}^{L}\left(T_{d}\right) \end{array}\right] \end{array}\right]$$

Each task shorter than the longest task was padded with zeros to equal the same length. The objective of reconstruction is closely analogous to finding a representation of *R* in a new basis *B*. This is accomplished as an optimization problem to find the elements of column vector *C* which most closely satisfies:

(6) R=BC

In this case, *B* will be made up of temporal offsets and dilations of the synergies defined by *S_m_*. *B* is formed by first transposing the downsampled version of *S_m_*. For notation, let *S(m)* be a J⋅*T_s_* element column vector containing synergy *m* and let [0] be a null column vector of *J* elements long. The first *T_d_ − T*_max_ columns of *B* are:

$$B_{D}^{m}=\left[\begin{array}{cccc} S\left(m\right) & \left[0\right] & & \left[0\right]\\ \left[0\right] & S\left(m\right) & & \left[0\right]\\ \left[0\right] & \left[0\right] & \ldots & \left[0\right]\\ \vdots & \vdots & & \vdots \\ \left[0\right] & \left[0\right] & & S\left(m\right) \end{array}\right]$$

where *m* = *1* is the synergy number, *D* = 0 identifies an undilated synergy (1, 2, or 3 indicating the first, second, or third synergy dilation), and *T*_max_ is the length of the undilated synergy.

Next, the synergy is dilated by interpolating *S(m)* to be some proportion of the difference between testing task length and synergy length. In this study, we dilate each synergy to be longer than the original synergy by 25, 50, and 75% of the difference in task and synergy sample length. Each dilation, *S_D_(m)*, is used to form the next *T_d_ − T_m,D_* columns of *B*, where *T_m,D_* is the number of samples in the *D*th dilations of the *m*th synergy. This is done by forming each column as a sequential temporal offset of *S_D_(m)* as done above. This process is repeated for each dilation of each synergy, resulting in a *B* matrix, which contains every temporal offset of every dilation examined of the first *M* synergies:

$$B=\left[B_{0}^{1}\;\;\ldots \;\;B_{3}^{1}\;\;\ldots \;\;B_{0}^{M}\;\;\ldots \;\;B_{3}^{M}\right]$$

*l*_1_-norm minimization was used to sparsely select values for *C* which satisfy the following optimization problem:

$$\text{Minimize }∥C{∥}_{1}+\frac{1}{\lambda }∥\mathrm{BC}-R{∥}_{2}^{2}$$

where ||⋅||_1_ and ||.||_2_ represent the *l*_1_- and *l*_2_-norms, respectively, and λ is a regulation parameter. Since the columns of *B* represent each synergy in different temporal offsets and dilations, the elements of *C* serve as recruitment weights for a synergy at a particular instant of time. The reconstructed task profiles, $\;\tilde{\;R}$, can be generated by multiplying *B⋅C*:

$$R\approx \;\tilde{\;R}=\mathrm{BC}$$

The error between the measured and reconstructed profiles across all joints *J* = *8* is computed for each task, *l* using

(7) $$e_{l}=\frac{\sum {\;}_{j=1}^{J}\sum {\;}_{t=0}^{T_{l}}{\left(r_{j_{j}}^{l}(t)-r_{j}^{l}(t)\right)}^{2}}{\sum {\;}_{j=1}^{J}\sum {\;}_{t=0}^{T_{l}}r_{j}^{l}{\left(t\right)}^{2}}$$

## Results

### Extraction of Synergies Using PCA

**Figure 2:**
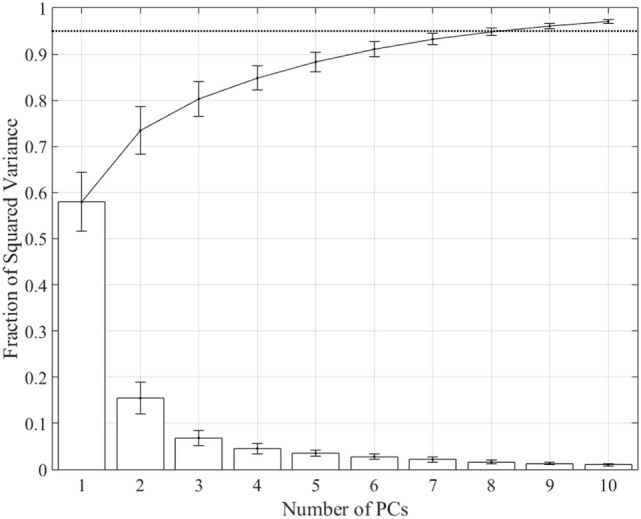
**Fraction of variance accounted for by each synergy (bars) and total from 1 to *n* synergies (line)**. The first synergy accounts for 57.98 ± 6.35% of variance, the first six synergies account for 91.05 ± 1.69%, and the first eight synergies account for 94.82 ± 0.85%. Dotted line shows 0.95 threshold.

Spatiotemporal kinematic synergies were computed for each subject using PCA. Figure 2 shows the squared variance for each synergy along with the fraction of the sum of squared variance averaged across subjects. Derived synergies were 2.14 ± 0.29 s long, ranging from 1.67 to 2.53 s. The first synergy accounts for 57.98 ± 6.4% of total variance, while the first six synergies account for 91.05 ± 1.7% and the first eight account for 94.82 ± 0.85%.

**Figure 3:**
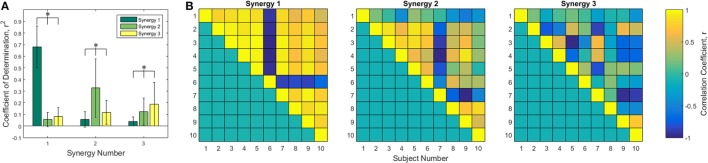
**Correlation analysis of the first three synergies**. **(A)** Pearson coefficient of determination, *r*^2^, averaged across the 45 unique combinations between the specified synergies of each of 10 subjects. Statistical differences found using one-way ANOVA tables with α = 0.05 with Tukey *post hoc* tests. Comparison of synergy 1 to synergy 1 and synergy 1 to synergies 2 and 3, among all pairings of synergy 2, and among all pairings of synergy 3 yielded significant differences. **(B)** Correlation coefficient, *r*, between each subject for synergies 1, 2, and 3. Only unique pairings of subjects are shown using the upper triangle matrices. Synergy 1 appears highly positively correlated except for subject 6, who is highly negatively correlated.

The first three of each subject’s synergies were compared to each other using Pearson’s correlation coefficient. Synergies were put into the column form discussed in the Section “Reconstruction” and compared. Figure 3A shows the Pearson’s *r*^2^ averaged across subject comparisons for each combination of the first three synergies, leading to 90 unique comparisons for each synergy pair. Statistically significant differences were found in each of the three groups by one-way ANOVA tables, α = 0.05. All comparisons between synergy 1, 2, and 3 were statistically significant (*p* ≪ 0.005 for all). Tukey *post hoc* tests were computed to determine specific differences. As expected, the correlation between synergy 1 and synergy 1 was greater than the correlation between synergies 1 and 2 and between synergies 1 and 3. Correlations between synergies 1 and 2 and between synergies 1 and 3 were not found to be different from each other. All *r*^2^ values are statistically different for the synergy 2 and 3 comparisons. A closer examination of the synergy correlations was conducted using Pearson’s correlation coefficient, *r*. Figure 3B is a color-scale grid displaying the comparison between synergy one, two, and three between each pair of subjects. Synergy 1 appears positively correlated across all subjects except subject 6, who appears to have a strong negative correlation. Synergy 2 appears more mixed: there exist some positive/negative correlations that contribute to the overall mean *r*^2^ of 0.3593 ± 0.2652, although subjects 1 and 10 have statistically insignificant mean *r* values at α = 0.05 of 0.0504 ± 0.265 (*p* = 0.5841) and 0.0594 ± 0.4582 (*p* = 0.7074), respectively.

**Figure 4:**
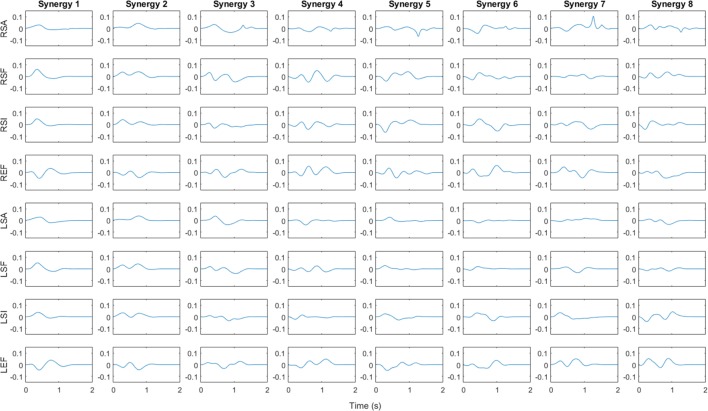
**Angular velocity profile for first eight synergies of subject 6**. Vertical axis is unitless velocity since synergy is unscaled. Rows are labeled with letters indicating the side (“R” for right or “L” for left), the joint (“S” for shoulder or “E” for elbow), and the rotation (“A” for abduction in positive direction, “F” for flexion in positive direction, “I” for internal rotation in positive direction) for each DoF. Synergy 1 and 2 involve flexion and internal rotation of shoulder along with extension of elbow, implying a reaching motion, whereas synergy 3 involves extensions at the shoulder and flexion at the elbow.

Figure 4 shows the synergy velocity profiles for the first eight synergies of subject 6. Each row corresponds to a joint labeled with a letter indicating the side (“R” for right or “L” for left), the joint (“S” for shoulder or “E” for elbow), and the rotation (“A” for abduction, “F” for flexion, “I” for internal rotation). Since the synergy is unscaled, the *y* axis is a unit-less velocity amplitude, while the *x* axis is time in seconds. For subject 6, the first synergy consists of bilateral shoulder flexion, abduction, and internal rotation, which suggests a forward reaching motion. The elbows both show a small initial flexion, perhaps as the subject raises their arms from the rest posture, followed by an extension motion as they complete the reach. Synergy 2 behaves similarly, except shoulder abduction is delayed relative to synergy 1 and shoulder flexion and internal rotation are executed for a longer period of time. As discussed above, synergy 3 is relatively uncorrelated among subjects. For subject 6, synergy 3 involves slight shoulder flexion followed by extension and adduction with an external rotation. The elbows appear to flex slightly, extend, then flex again.

**Figure 5:**
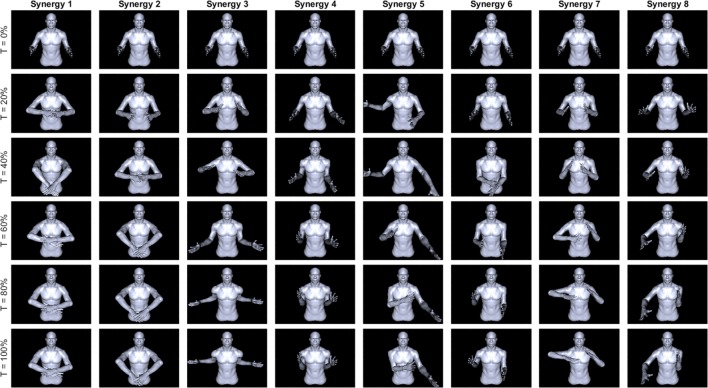
**Posture visualization for first eight synergies of subject 6 (columns) over six normalized time instances (rows)**. Position at *T* = 0% is the subject’s position averaged across the first 50 samples of all tasks. Synergies were integrated up to each time point and multiplied by a gain such that normal joint range of motion is not violated. Mirror symmetry between left/right arms can be seen in the first three synergies whereas synergies 4–8 have asymmetric motions. Subject’s non-dominant hand tends to go to a single position and hold steady while the dominant hand appears to move continuously.

In order to better visualize the other synergies for subject 6, each profile was integrated, multiplied by a gain, and added to the average starting joint angles of a particular subject. The gain was chosen such that the resulting angular profile remains within natural range of motion throughout the whole path. Figure 5 shows a virtual mannequin posed at the resulting postures for six normalized time points. Subject 6, with a negatively correlated synergy 1, has a distinct reach-and-grasp motion. The downward dip of both hands observed at *t* = 0.4, or nearly halfway through the motion, could roughly correspond to the end of the reach phase and the beginning of object grasp and manipulation involving picking up an object. Note that subject 6’s first synergy is strongly negatively correlated with the rest of the subjects, so synergy 1 for most of the subjects involves outward extension of the arms, similar to synergy 3 in Figure 4. Synergy 2 was similar, except it involved a reach-and-grasp motion instead of reach and manipulate/pick up. The shoulder motions observed in the synergy 3 profiles clearly result in an overall bilateral extension movement, with the arms spanning outward behind the back. Synergy 4 appears similar to picking up and holding a box at chest level.

**Figure 6:**
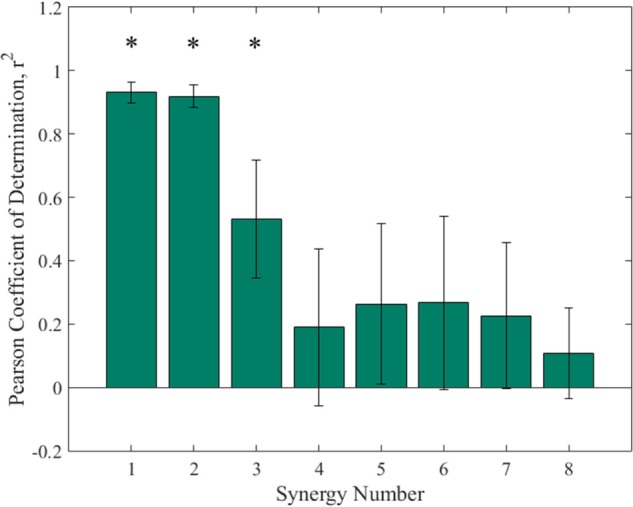
**Pearson’s coefficient of determination comparing right arm joints to left arm joints of the each synergy**. One-way ANOVA and Tukey *post hoc* show that synergy 1 and synergy 2 each had significantly higher coefficients of determination than synergies 4–8, while the coefficient for synergy 3 was significantly higher than synergy 4 and 8. Statistical difference found in first three synergies implies asymmetric motion between left and right arms in higher order synergies.

The higher order synergies shown in Figure 5 appear to show a level of handedness, with the left hand tending to go to a certain position and holding while the right hand moved in various profiles through the duration of each synergy. Figure 6 shows the Pearson coefficient of determination, *r*^2^, averaged across subjects comparing joints 1–4 (right arm) to joints 5–8 (left arm) of all eight synergies. A one-way ANOVA (α = 0.05) was performed with Tukey *post hoc* tests to establish significant differences (ANOVA *p* ≪ 0.005). Synergies 1 and 2 were each significantly more correlated between left/right than Synergies 4–8, and Synergy 3 was more correlated between left/right than Synergies 4 and 8.

### Task Reconstruction

**Figure 7:**
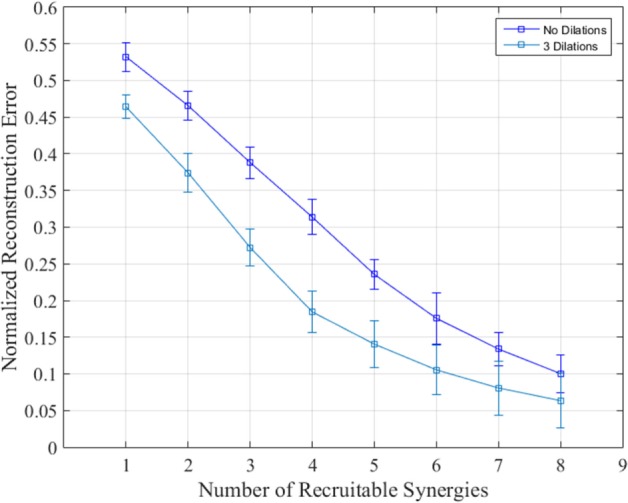
**Normalized reconstruction error when recruiting from synergies 1–8 with and without dilations**. The reconstruction error for each synergy was averaged over degrees of freedom, subjects, and tasks. Dilated synergies were longer than undilated synergies by 25, 50, and 75% of the difference between minimum reconstruction task length and the synergy length. SDs are across subjects and tasks.

Reconstruction was carried out using up to eight synergies given the PCA results above and the 8-DoF nature of the bilateral arm model presented. Tasks were reconstructed with and without dilations. Figure 7 shows the mean reconstruction error calculated using Eq. 7 and averaged across degrees of freedom, tasks, and subjects using no dilations (blue) and dilations at 25, 50, and 75% of task/synergy length difference. Synergies 1–4 show a nearly linear decrease in normalized error in both cases; each subsequent synergy begins showing less of an improvement. Recruiting the first six synergies yielded a normalized reconstruction error of 0.1757 ± 0.0347 and 0.104 ± 0.0161 for no dilations and three dilations, respectively. Ultimately the error reduces down to 0.062 ± 0.0098 by synergy 8.

**Figure 8:**
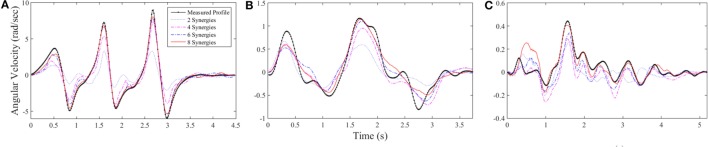
**Three examples of reconstruction progression for left shoulder abduction**. **(A)** Repetition 2 of task 27 for subject 8, the best-performing reconstruction with error across all joints of 2.22%. **(B)** Repetition 2 of task 24 for subject 5, error of 2.4%. **(C)** Repetition 1 of task 22 for subject 10, error of 7.3%. The early reaching phases of tasks were typically not as accurately reconstructed as the later manipulation phases.

Figure 8 shows examples of the reconstruction progression for left shoulder abduction using dilations for several tasks, demonstrating a clear progression from two available synergies (blue dotted line) to 8 synergies (red line). Figure 8A shows that the best-performing task that was repetition 2 of task 27, pretend steering wheel counterclockwise for subject 8. Reconstruction error went from 74.2% using only the first synergy to 2.22% using the first eight. Figure 8B shows repetition 2 of task 24, open box, for subject 5. Reconstruction error for this task was 27.9% using the first synergy and 2.41% using the first eight. Figure 8C shows repetition 1 of task 22, knife and fork, for subject 10. Reconstruction error was 43.8% using the first synergy and 7.32% using the first eight.

The optimization algorithm appeared to handle cyclic profiles relatively well, whereas the initial and ending phases of the tasks would often be less accurate. Figures 8A,C show this quality within the first 1 s of the tasks. Reconstruction was able to capture the overall shape of the task shown in Figure 8B, but some of the finer, irregular motions were not captured.

Tables 5 and 6 show the integrated recruitment gain for subject 4 averaged across tasks during normalized time bins for each synergy without and with dilations, respectively. Synergies and their dilations were not allowed to be recruited beyond the time at which the last synergy and task sample would coincide. Time bins which would include “missing” recruitment gains were therefore omitted. Significant differences found using one-way ANOVA with α = 0.00089 (0.05 over 56 comparisons) between synergy recruitments with and without dilations are indicated in Table 5 as bolded and underlined with an asterisk. For subject 4, synergies 3 and 5 in time bin 2 were significantly more recruited in the no dilations reconstruction than in the dilations reconstruction (*p* ≈ 0 for both). No other synergies had significantly different recruitments.

**Table 5:** **Integrated recruitment weight without dilations for normalized time bins averaged across tasks for subject 4 (mean ± SD)**.

		Time bin							
		1	2	3	4	5	6	7	8–10
Synergy number	1	−26.57 ± 17.45	−11.33 ± 26.62	12.06 ± 34.19	−9.23 ± 18.79	−9.48 ± 26.38	7.96 ± 28.23	−0.72 ± 16.06	–
	2	9.44 ± 18.81	−4.75 ± 15.67	1.74 ± 10.06	0.37 ± 9.64	−4.33 ± 14.96	0.24 ± 8.66	0.64 ± 9.37	–
	3	4.98 ± 12.94	9.89 ± 12.61*	−3.08 ± 12.48	−0.35 ± 10.6	2.44 ± 9.28	−6.18 ± 14.18	1.14 ± 13.08	–
	4	8.03 ± 9.53	−0.01 ± 11.62	1.87 ± 9.43	−2 ± 8.59	1.7 ± 7.77	−0.88 ± 6.15	2.4 ± 9.47	–
	5	1.39 ± 13.96	−9.24 ± 11.7*	1.41 ± 11.23	4.93 ± 13.45	−4.74 ± 9.3	0.29 ± 8.04	2.05 ± 10.15	–
	6	6.42 ± 28.58	−17.88 ± 51.59	5.55 ± 28.93	10.64 ± 34.04	−14.79 ± 33.27	5.74 ± 20.07	8.41 ± 30.17	–
	7	−0.34 ± 9.14	−3.77 ± 15.21	0.8 ± 15.6	2.91 ± 16.71	−4.09 ± 13.76	5.93 ± 11.02	0.72 ± 14.61	–
	8	2.53 ± 24.7	−12.33 ± 33.39	−4.37 ± 37.26	16.78 ± 36.35	−13.31 ± 26.3	−3.19 ± 31.74	10.07 ± 28.91	–

*Statistical differences are marked in bold with an asterisk*.

**Table 6:** **Integrated recruitment weight including dilations (*D*_0_, *D*_1_, *D*_2_, *D*_3_) for normalized time bins averaged across tasks for subject 4 (mean ± SD)**.

			Time bin							
			1	2	3	4	5	6	7	8–10
Synergy number	1	*D*_0_	−19.75 ± 18.81*	3.53 ± 14.85	5.04 ± 17.22	−1.97 ± 5.45	0.78 ± 4.39	−0.47 ± 11.58	−2.28 ± 6.33	–
		*D*_1_	−4.79 ± 5.17	1.06 ± 4.05	−1.49 ± 7.14	−0.35 ± 5.84	0.85 ± 6.59	–	–	–
		*D*_2_	−4.79 ± 5.17	1.06 ± 4.05	−1.49 ± 7.14	–	–	–	–	–
		*D*_3_	−3.1 ± 7.41	–	–	–	–	–	–	–
	2	*D*_0_	1.42 ± 14.16	0.27 ± 0.89	2.09 ± 8.09	−0.16 ± 2.02	0.46 ± 1.36	−0.06 ± 6.2	1.36 ± 4.4	–
		*D*_1_	−0.83 ± 9.06	0.37 ± 1.59	−0.54 ± 2.59	−0.19 ± 1.77	1.54 ± 3.9	–	–	–
		*D*_2_	−1.61 ± 5.17	1 ± 4.05	−3.55 ± 7.14	–	–	–	–	–
		*D*_3_	−3.04 ± 7.77	–	–	–	–	–	–	–
	3	*D*_0_	0.5 ± 1.71	−0.42 ± 1.53	−0.03 ± 1.2	0.07 ± 1.16	−0.7 ± 1.41	0.57 ± 2.06	0.67 ± 2.12	–
		*D*_1_	0.48 ± 1.75	−0.42 ± 1.53	−0.03 ± 1.2	0.07 ± 1.16	−0.7 ± 1.4	–	–	–
		*D*_2_	0.56 ± 5.49	−0.41 ± 1.31	−0.15 ± 1.42	–	–	–	–	–
		*D*_3_	5.82 ± 12.58	–	–	–	–	–	–	–
	4	*D*_0_	3.06 ± 8.86	−0.32 ± 4.15	−0.8 ± 3.01	0.9 ± 2.07	−0.62 ± 2.47	−0.06 ± 2.55	0.85 ± 1.35	–
		*D*_1_	2.63 ± 5.85	−0.37 ± 3.09	−0.87 ± 3.38	0.99 ± 2.32	−0.51 ± 2.39	–	–	–
		*D*_2_	1.52 ± 8.97	0.3 ± 4.82	−0.76 ± 3.27	–	–	–	–	–
		*D*_3_	1.48 ± 5.9	–	–	–	–	–	–	–
	5	*D*_0_	−1.45 ± 2.48	0.09 ± 1.69	−0.41 ± 1.31	−0.52 ± 1.37	−0.05 ± 0.81	0.34 ± 2.93	0.03 ± 1.35	–
		*D*_1_	−1.46 ± 2.49	0.09 ± 1.69	−0.41 ± 1.31	−0.53 ± 1.38	−0.04 ± 0.76	–	–	–
		*D*_2_	−2.96 ± 7.54	−1.15 ± 3.6	0.55 ± 3.75	–	–	–	–	–
		*D*_3_	−0.28 ± 5.97	–	–	–	–	–	–	–
	6	*D*_0_	2.31 ± 22.83	−0.19 ± 13.51	4.05 ± 17.21	−0.47 ± 16.69	−2.68 ± 13.1	3.54 ± 13.58	−2.15 ± 8.03	–
		*D*_1_	3.65 ± 11.23	−2.2 ± 7.98	1.2 ± 2.92	−0.91 ± 5.01	−1.75 ± 7.8	–	–	–
		*D*_2_	−5.32 ± 15.15	−0.8 ± 5.65	6.17 ± 14.32	–	–	–	–	–
		*D*_3_	−3.85 ± 9.58	–	–	–	–	–	–	–
	7	*D*_0_	1.65 ± 7.15	−1.16 ± 3.6	0.97 ± 4.61	0.64 ± 8.32	−1.35 ± 6.54	2.17 ± 5.3	−3.59 ± 7.96	–
		*D*_1_	2.66 ± 14.93	0.53 ± 17.11	−7.03 ± 10.51*	2.07 ± 9.08	−1.86 ± 5.2	–	–	–
		*D*_2_	−0.38 ± 13.73	3.37 ± 8.03	−0.52 ± 5.7	–	–	–	–	–
		*D*_3_	2.06 ± 6.86	–	–	–	–	–	–	–
	8	*D*_0_	25.04 ± 58.14*	−1.4 ± 10.05	−10.33 ± 25.53*	2.32 ± 11.15	−1.02 ± 4.59	−7.8 ± 21.86	1.46 ± 6.18	–
		*D*_1_	−0.73 ± 3.85	−1.03 ± 3.49	0.66 ± 2.72	−0.67 ± 1.38	0.88 ± 3.1	–	–	–
		*D*_2_	−11.03 ± 28.59	−2.25 ± 10.4	9.63 ± 22.25	–	–	–	–	–
		*D*_3_	−8.24 ± 25.78	–	–	–	–	–	–	–

*Statistical differences are marked in bold with an asterisk*.

Table 6 shows significant differences in recruitment between dilations of each synergy in each time bin, found using one-way ANOVA tables with α = 0.00125 (0.05 over 40 comparisons) and Tukey *post hoc* tests. Differences are bolded and marked with an asterisk. The undilated synergy 1 was significantly more recruited than all dilations in time bin 1 (*p* ≈ 0). The undilated synergy 8 was significantly more recruited than dilation 2 and 3 in time bin 1 (*p* ≪ 0.00125). The first dilation of synergy 7 was significantly more recruited than the un-dilated synergy in time bin 3 (*p* ≈ 0). The undilated synergy 8 was significantly more recruited than the second dilation in time bin 3 (*p* ≪ 0.00125), although the absolute value of their recruitments likely would not be different.

A similar analysis was performed after pooling all subjects. Significant differences in recruitment of an undilated synergy including and excluding dilations were found across tasks and subjects using ANOVA with α = 0.0013 (α = 0.05 for 40 comparisons). Synergy 1 was significantly less recruited in time bins 1 and 2 when dilations were included in reconstruction (*p* ≈ 0). Synergy 8 was significantly more recruited in time bin 1 with dilations compared to without dilations (*p* ≪ 0.0013). Differences in recruitment among dilations of each synergy in each time bin were found across tasks and subjects using ANOVA tables with α = 0.00156 (α = 0.05 for 32 comparisons). The undilated synergy 1 in time bin 1 is significantly more recruited than the dilated versions (*p* ≈ 0). The undilated and first dilation of synergy 5 are more recruited than dilation 3, but this difference was not statistically significant (*p* = 0.0034). Dilation 4 of synergy 7 was recruited significantly more than the undilated, first, and second dilation in time bin 1 (*p* ≪ 0.001). The undilated synergy 8 was statistically different from the first and second dilation in time bin 1 (*p* = 0.0015).

## Discussion

Previous work in implementing postural synergies in robotic control systems (see Introduction) aims to create a simplified control scheme, which can control high-dimensional systems with a reduced number of control inputs or actuators. The present study expands on this work by evaluating time-varying spatiotemporal synergies defined for both shoulder and elbow joints. Furthermore, we believe a key question for robotic systems (such as prosthetics and other assistive devices) meant to perform ADL, is how capable such systems are of reproducing ADL-like motion. Most experiments evaluate performance via endpoint variables such as task success rate, task completion time, or endpoint accuracy. This work attempts to evaluate such a system based on its ability to replicate ADL-like kinematics measured from reach-and-grasp experiments. This representation gives a more complete representation of the state of the system through the entire motion being performed. We present the optimal kinematic performance of time-varying synergies by sparsely optimizing the recruitment of synergies in time and amplitude and also introduce temporal dilations as discussed later.

Our results indicate that although the first three synergies can account for up to 80% of variance in training data, replicating the actual kinematics of ADL tasks could require the use of higher order synergies. Using two, three, or even four synergies as in previous literature could still be placing an absolute optimal kinematic accuracy limit of over 30% error in angular velocity, as shown in Figure 7. In the context of robotic control and assistive robotic interfaces, our results are reminiscent of the tradeoff between the optimal performance and complexity of control. An autonomous system may be able to incorporate higher order synergies in order to finely coordinating two arms, whereas an assistive system meant to be used by a person would quickly become too complex to use. These results may be improved on by experimenting with training task composition, dividing joints into different subgroups, or several other approaches which were not examined here.

Beyond simplifying control schemes, synergies have also been proposed as an avenue to make robotic systems exhibit more humanoid behaviors with biomimetic control. Robots designed to interact and cooperate with humans are deemed more esthetically pleasing when exhibiting anthropomorphic motion and are believed to be safer since human-like motions are more readily predictable than robotic ones (Duffy, 2003; Hegel et al., 2008). Control schemes aimed at anthropomorphizing robotic motions through the use of synergies derived from reach-and-grasp tasks have been carried out by Liarokapis et al. (2015) and reviewed by Santello et al. (2016). Whereas this work leveraged postural synergies, our results indicate that time-varying synergies could be used to produce more accurate replications of human motion. It is possible that the reconstruction method applied here could be performed on non-anthropomorphic trajectories to generate a near-natural approximation of a synthetictrajectory.

The recent trend of mechanically embedding synergies into robotic systems may also be extended to time-varying principal components. The SoftHand, for example, uses a compliant structure actuated by a single principal component to achieve various grasps (Catalano et al., 2014). Implementing the complex motions described in Figure 4 may be achieved by software-generated temporal postural synergies coupled with a mechanical design which together introduce non-linearities. These time-varying synergies in bilateral arm movements can be easily integrated in upper limb prosthetics and rehabilitation devices to render natural movements.

Our results could also have implications outside of strict robotic control. The symmetric nature of the first two synergies between the left and right arms led to an examination of the similarities between the left and right joint angular velocities of synergies 1–8 for each subject. These results indicate that lower order synergies tended to have similar profiles between the left and right arms, whereas higher order synergies were dissimilar. The lower order synergies seem to provide broad reaching motions, while higher order synergies appear to fine-tune the motion to suit particular manipulations required by a task.

The asymmetries in higher order synergies could contain information related to the handedness of the subject. Observationally, the postural visualization of the higher order synergies in Figure 3 appears to show the left arm maintaining stable positions and motions, while the right arm follows a more complex path. This result is in line with previous work done on bimanual upper limb control, and a more detailed kinematic analysis of bilateral kinematic synergies may also be conducted to explore this link. Sainburg and Kalakanis (2000) and Bagesteiro and Sainburg (2002) found significant differences in hand path curvature and torque efficiency between dominant and non-dominant hands; the same group found advantages in the dominant arm for speed and directional control, whereas the non-dominant arm was specialized for accurate position control through increased limb impedance (Wang and Sainburg, 2007). Recently, Yokoi et al. (2014) conducted bimanual lever tasks in which the non-dominant arm operated in an artificial force field. They were able to demonstrate that the non-dominant limb was more adaptable to the force field when trained with a specific motion direction of the dominant arm, but that altering the dominant-side target location led to a decrease in performance of the non-dominant arm. In this paper, a model using movement primitives to approximate motor learning of the non-dominant arm relative to dominant arm dynamics was able to replicate this effect, supporting the idea that bilateral synergies may be used by the central nervous system to produce motion.

The synergies are hypothesized to be abstract representations of motion encoded in the sensorimotor system of the brain, which can be combined to form the complex motions required to execute ADL tasks. The actual generation and execution of complex upper limb movements is a neural process that still has much to be discovered. The original drive or goal which the sensorimotor system acts on has long been believed to be the limbic system (Brooks, 1986). The abstract goals produced by the limbic system are processed by the association cortex that then produces a plan for motion. This information is passed to the projection system, composed of the sensorimotor cortex, cerebellum, and basal ganglia, which converts the “trajectory” to a series of commands which can be passed to the spinal cord. This process is dictated by sensory feedback, developmental history, and a sense of the dynamics of the body (Brooks, 1983). The internal dynamic model has been hypothesized to be composed of both feedforward and feedback components, aimed at minimizing error while still allowing for quick responses. The existence of either of these models or the combination thereof is still much debated, with evidence pointed either way (Wolpert et al., 1998). As mentioned earlier, the studies conducted by Viviani and Terzuolo (1980) imply the projection system’s ability to dilate motion building blocks in time. Single impulses at specific interneuronal sites result in coactivation of multiple muscles, as shown by Tresch et al. (1999) and Saltiel et al. (2001), and each muscle generates torque around multiple degrees of freedom in the hand and arm (Santello et al., 2013). This torque is influenced by numerous anatomical parameters which are unique to each individual, as is the resulting motion of the limb (Buchanan et al., 2004). Visual, proprioceptive, haptic, and numerous other sensory signals are then returned to the brain and integrated into motion planning.

Task reconstruction as performed in this study attempts to model the neural process described above computationally. The measured training task can be viewed as the motion plan output by the associative cortex. The synergies derived from the rapid tasks serve as the “history” programmed into the projective system which dictates how the brain converts planned motion to spinal cord commands. This would be analogous to a feedforward component of cerebellar processing discussed by Wolpert et al. (1998): given a certain output from the brain, there is expected to be some dynamic response observed at the arms that can take on an arbitrary pattern across joints. Dilations of the synergies are also provided which attempts to mimic the time-scaling ability of the basal ganglia and cerebellum. *l*_1_-norm minimization serves the role of the projection system by using synergies and their dilations to convert from a desired motion to a time-series of gains, which can be viewed as the time-series of signals being sent to the spinal cord. The neuromuscular and physiological construction of the limbs is approximated by multiplying the synergies by this time-series of gains, producing the final motion.

The time-series of gains generated by *l*_1_-norm minimization could give some insights into how useful temporal dilations of synergies could be in motion production. Table 5 shows that recruitment of synergy 1 is reduced when including dilations in reconstruction for the first two time bins. Synergy 8 had a larger recruitment in the first time bin with dilations compared to without. Table 6 shows that the only statistical difference between recruitment of dilations is in time bin 1 for synergies 1, 7, and 8. The undilated synergy 1 was significantly more recruited than the dilations, dilation 3 was the most recruited for synergy 7, and the undilated synergy was the most recruited for synergy 8. The most significant effect of including dilations in the recruitment process appears to be the reduction in recruitment of synergy 1 during task onset. Recruitment of the dilations of synergy 1 also appears to be relatively large compared to recruitment of the other synergy dilations.

The present experiment could be refined to further study dominant/non-dominant performance in bimanual ADL. The subject pool can be expanded to include left- and right-handed individuals, and tasks can be modified to selectively engage the left, right, or bilateral arms. The relative influence of dominant/non-dominant hemispheres on ipsilateral limb motion could be examined with a more focused data analysis by examining whether left or right side joints are better reconstructed for left- and right-handed subjects. Devising a way to “coax” reconstruction to replicate the structural asymmetry and increased influence of the dominant over the non-dominant hemisphere as opposed to *vice versa* (Snyder et al., 1995; Amunts et al., 1996; Ziemann and Hallett, 2001; Hayashi et al., 2008) could allow us to study the strength of this influence. The upper limb kinematic data recorded in this study may also be augmented with EMG and/or EEG signals.

## Conclusion

This paper presents a study of spatiotemporal kinematic synergies in bilateral arm movements during ADL tasks. These tasks were selected as a representation of the fundamental categories of bilateral motion (symmetric, asymmetric, coupled), with further work aimed at tailoring the task list to target specific aspects of sensorimotor processing. Derived synergies accounted for up to 94.82 ± 0.85% of variance and were demonstrated to reconstruct ADL tasks to within 6.2 ± 0.98% using the *l*_1_-norm minimization algorithm. The concept of temporal synergy dilations were incorporated to replicate movement processing found in the basal ganglia and cerebellum. Recruitment patterns were examined throughout task duration and found that the dilated version of a synergy was used equally as much as the undilated version of the same synergy in most time bins, with the beginning of the motion having the most difference. Potential uses of time-varying synergies to anthropomorphize robotic motion and inform mechanical construction were discussed. Interesting features of the synergies in the context of handedness corroborating others’ results were discussed. We believe that synergies will be instrumental in building next-generation biomimetic prosthetics and orthotics in the near future.

## Ethics Statement

The present study was conducted under IRB Approved Protocol # 2014-026/2015-022 at the Stevens Institute of Technology. All the subjects gave written informed consent in accordance with the Declaration of Helsinki.

## Author Contributions

MB designed the experiment, ran instrument calibrations, designed the kinematic model and data processing scripts (with inputs from IF and KP), executed the experiment, and wrote the manuscript. VP contributed to the data processing scripts, assisted with executing the experiment, and provided valuable feedback in writing the manuscript. RV set up the hypothesis and direction/scope of the study, provided invaluable feedback in experiment design, task selection, and data processing, coordinated subject recruitment, and provided key revisions and editing on the manuscript.

## Conflict of Interest Statement

The authors declare that the research was conducted in the absence of any commercial or financial relationships that could be construed as a potential conflict of interest. The reviewer MB and handling editor declared their shared affiliation, and the handling editor states that the process nevertheless met the standards of a fair and objective review.
